# The sonic hedgehog signaling pathway is reactivated in human renal cell carcinoma and plays orchestral role in tumor growth

**DOI:** 10.1186/1476-4598-8-123

**Published:** 2009-12-16

**Authors:** Valérian Dormoy, Sabrina Danilin, Véronique Lindner, Lionel Thomas, Sylvie Rothhut, Catherine Coquard, Jean-Jacques Helwig, Didier Jacqmin, Hervé Lang, Thierry Massfelder

**Affiliations:** 1 INSERM U682, Section of Renal Cancer and Renal Physiopathology, University of Strasbourg, School of Medicine, Strasbourg, 67085 France; 2 Hôpital de Mulhouse, Department of Pathology, Mulhouse, 68000 France; 3 Nouvel Hôpital Civil de Strasbourg, Department of Urology, Strasbourg, 67091 France

## Abstract

**Background:**

Human clear cell renal cell carcinoma (CRCC) remains resistant to therapies. Recent advances in Hypoxia Inducible Factors (HIF) molecular network led to targeted therapies, but unfortunately with only limited clinical significance. Elucidating the molecular processes involved in kidney tumorigenesis and resistance is central to the development of improved therapies, not only for kidney cancer but for many, if not all, cancer types. The oncogenic PI3K/Akt, NF-kB and MAPK pathways are critical for tumorigenesis. The sonic hedgehog (SHH) signaling pathway is crucial to normal development.

**Results:**

By quantitative RT-PCR and immunoblot, we report that the SHH signaling pathway is constitutively reactivated in tumors independently of the von Hippel-Lindau (VHL) tumor suppressor gene expression which is inactivated in the majority of CRCC. The inhibition of the SHH signaling pathway by the specific inhibitor cyclopamine abolished CRCC cell growth as assessed by cell counting, BrdU incorporation studies, fluorescence-activated cell sorting and β-galactosidase staining. Importantly, inhibition of the SHH pathway induced tumor regression in nude mice through inhibition of cell proliferation and neo-vascularization, and induction of apoptosis but not senescence assessed by in vivo studies, immunoblot and immunohistochemistry. Gli1, cyclin D1, Pax2, Lim1, VEGF, and TGF-β were exclusively expressed in tumors and were shown to be regulated by SHH, as evidenced by immunoblot after SHH inhibition. Using specific inhibitors and immunoblot, the activation of the oncogenic PI3K/Akt, NF-kB and MAPK pathways was decreased by SHH inhibition.

**Conclusions:**

These findings support targeting SHH for the treatment of CRCC and pave the way for innovative and additional investigations in a broad range of cancers.

## Background

Renal cell carcinoma (RCC) is the most lethal urologic tumor and the sixth leading cause of cancer deaths in Western countries. Each year, around 200,000 patients are diagnozed with this malignancy resulting in approximately 100,000 deaths, and its incidence is increasing steadily [1,2]. RCC is represented by 80% by clear cell RCC (CRCC), originating from the renal proximal tubule. RCC is resistant to radio-, hormono-, and chemotherapy, and immunotherapy is effective in only 15% of selected patients [3]. The recent development of anti-angiogenic strategies based on small molecule tyrosine kinase receptor inhibitors lead to the approval of sunitinib or sorafenib as first-line therapy for RCC [2-5].

So far the best known oncogenic signal in human CRCC is constituted by the von Hippel-Lindau (VHL) tumor suppressor gene and hypoxia-induced factors (HIFs). Inherited and sporadic forms of CRCC are associated with inactivation of the VHL gene [6,7]. In hypoxic conditions, or when the VHL gene is defectuous as it is the case in 60% of CRCC, HIFs-α are stabilized allowing the expression of a large panel of target genes involved in growth, motility, metabolism and angiogenesis such as vascular endothelium growth factor (VEGF), tumor growth factors (TGFs), parathyroid hormone-related protein (PTHrP), glucose transporters and transferrin [1,7], all shown to contribute to CRCC tumorigenesis.

Additional oncogenic events are required for CRCC formation, and such concept has been clearly evidenced by molecular and genetic approaches [8]. We and others have shown that the proliferative and survival signaling pathways such as the PI3K/Akt, NF-κB and MAPK pathways are constitutively activated and turned towards tumor growth in human CRCC [9-11]. The idea that tumors hijack for their own growth signaling pathways involved in normal development is emerging. In human CRCC, this is the case for at least the Pax2 and 8 transcription factors and Notch signalling [12,13].

The hedgehog pathway is critical for embryonic and postnatal organ and tissue development, including the kidney. The sonic hedgehog (SHH) signaling pathway has also been shown to be dysregulated in pancreatic and colorectal cancers and melanomas [14], resulting in the induction of the expression of numerous target genes that regulate cell proliferation, cell differentiation, cell death, extracellular matrix interactions, and angiogenesis [15]. The SHH pathway interacts with various oncogenic pathways including the PI3K/Akt, the NF-κB, the MAPK pathways and the Notch pathway, another important developmental pathway. Interestingly, these pathways have been shown by us and others to be critical for human CRCC tumorigenesis [9-13]. To date and to our knowledge no studies have been conducted to assess the importance of the SHH pathway in human CRCC tumorigenesis and that was the purpose of the present study.

We found that the SHH signalling pathway is reactivated in human CRCC and that it converges to various oncogenic pathways to orchestrate tumor growth. In addition, we identified various Gli1 targets some never previously described such as Smo and the transcription factor Lim1 that is also necessary for normal kidney development.

## Results

### SHH signaling pathway components are constitutively expressed in human CRCC cells independently of VHL expression

**Figure 1:**
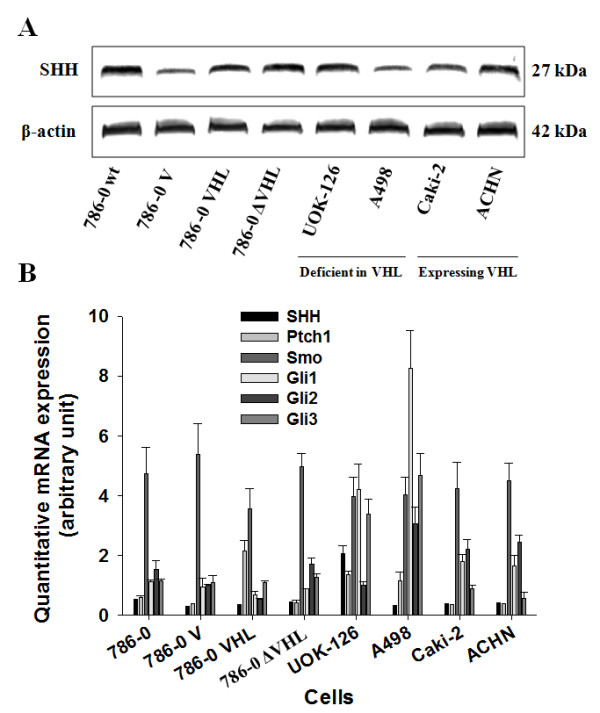
**All the SHH signaling pathway components are expressed in human CRCC cells independently of VHL expression**. (*A*) Western blot analysis of the SHH ligand in human CRCC cell lysates incubated with antibodies against human SHH ligand and corresponding β-actin. The expression of the ligand was assessed in 786-0 cells either untransfected (786-0 wt) or transfected with the vector alone (786-0 V), the full-length human VHL cDNA (786-0 VHL) or truncated inactive VHL cDNA sequence (786-0 ΔVHL), as well as in a panel of human CRCC cell lines either deficient in VHL expression (Deficient in VHL) or expressing VHL (Expressing VHL). The gels shown are representative for at least 3 independent experiments. (*B*) Quantitative gene expression of SHH ligand, of Ptch1 and Smo receptors, and of the Glis transcription factors (Gli1, Gli2 and Gli3) in the same panel of cells depicted in (*A*). Results are shown as mean ± SEM, n = 4.

The SHH ligand expression was detected in untransfected 786-0 cells (wt) and in 786-0 cell either untransfected or transfected with the various VHL constructs, as well as in a panel of human CRCC cell lines expressing or not VHL (Figure 1A).

All the components of the SHH signaling pathway, i.e SHH ligand, Ptch1, Smo and the downstream transcription factors Glis were expressed in all cells (Figure 1B). In all cases, except A498 cells, Smo was the highest expressed component. There was no difference in expression depending on the VHL status (Figure 1B).

Thus, the SHH signaling pathway is constitutively expressed and activated in tumor cells and independently of VHL expression.

### SHH signaling pathway components are constitutively reexpressed in human CRCC tumors

**Figure 2:**
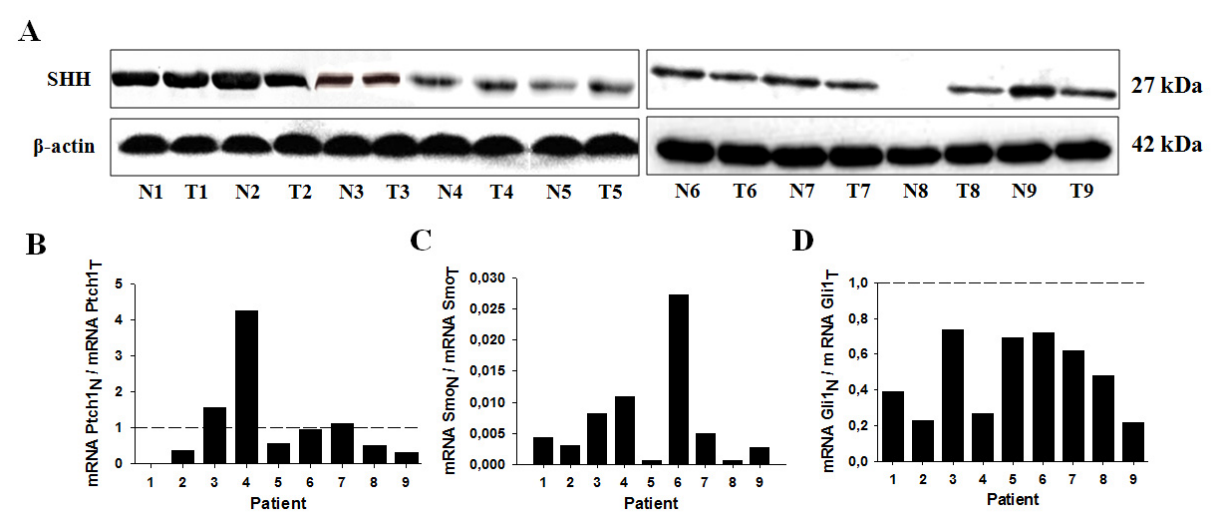
**All the SHH signaling pathway components are expressed in human CRCC tumors**. (*A*) Western blot analysis of the SHH ligand in 9 human tumors (T1 ...) and normal corresponding tissues (N1 ...) lysates incubated with antibodies against human SHH ligand and corresponding β-actin. The gels shown are representative for at least 3 independent experiments. (*B*) Quantitative gene expression of Ptch1 and Smo receptors, and of the Gli1 transcription factor in the same normal/tumoral tissue pairs shown in (*A*). Results are representative of 4 independent experiments.

The SHH ligand was detected in all tumor samples as well as in normal corresponding tissues for all stages except for patient 8 (T8) where SHH was undetectable in normal tissue (N8) (Figure 2A).

The Ptch1 receptor ratio was very variable from one N/T sample pair to another being either less expressed in normal tissue, equally expressed in tumors and normal tissues or higher in normal tissue (Figure 2B). Interestingly, the expression of the Smo receptor was considerably higher (300 to 1,000 fold increase) in tumors compared to normal corresponding tissues for all N/T pairs tested (Figure 2C). The expression of the Gli1 transcription factor was also increase about two- to five- fold in tumors compared to normal corresponding tissues (Figure 2D).

Taken together these results show that the SHH signaling pathway is active in tumors compared to normals.

### SHH signaling pathway inhibition decreases human CRCC cell proliferation independently of VHL expression

**Figure 3:**
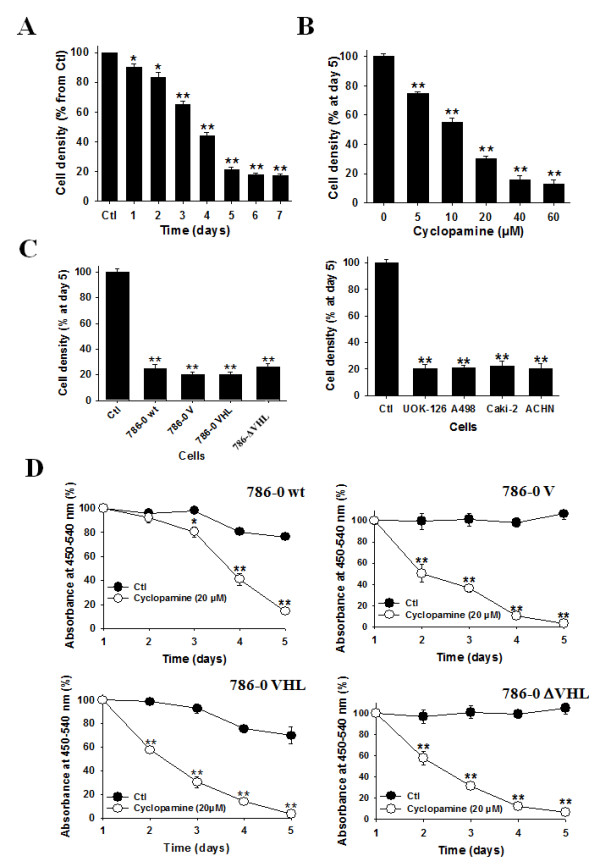
**The inhibition of the SHH signaling pathway decreases human CRCC cell proliferation**. (*A*) Human 786-0 cells were treated in control (Ctl) or with cyclopamine (Cyclopamine, 20 μM) and cells were counted each day. Results are shown as mean ± SEM, n = 6; *, *P *< 0.05; **, *P *< 0.01 from Ctl. (*B*) Human 786-0 cells were treated for 5 days in control (Ctl) or with cyclopamine (Cyclopamine) and adherent cells were counted. Results are shown as mean ± SEM, n = 6; **, *P *< 0.01 from 0 (without cyclopamine). (*C*) Our panel of human CRCC cells either deficient or expressing VHL were treated treated for 5 days in control (Ctl) or with cyclopamine (Cyclopamine, 20 μM) and adherent cells were counted. Results are shown as mean ± SEM, n = 6; **, *P *< 0.01 from Ctl (without cyclopamine) that was set to 100%. (*D*) Human 786-0 wt, 786-0 V, 786-0 VHL and 786-0 πVHL cells were analyzed for BrdU incorporation after treatment in control (Ctl) or with cyclopamine (Cyclopamine, 20 μM), for the indicated periods of time. Results are shown as mean ± SEM, n = 6; *, P < 0.05; **, *P *< 0.01 from corresponding Ctl.

Cyclopamine at 20 μM decreased cell proliferation by up to 80% after 5 days of treatment (Figure 3A). The effect of the inhibitor was concentration-dependent with a maximal effect of 90% inhibition of cell proliferation at 40 μM at day 5 (Figure 3B). For the rest of the experiments we choose tu use cyclopamine at 20 μM, a concentration near the IC50 on cell growth.

The efficacy of the inhibitory effect of cyclopamine was not dependent on the VHL status and was identical also in our panel of human CRCC cell lines (Figure 3C).

The effect of cyclopamine on cell growth was due in a large part to inhibition of cell proliferation as assessed by BrdU incorporation studies in 786-0 wt cells, in 786-0 V, 786-0 VHL and 786-0 ΔVHL (Figure 3D), with a maximal inhibitory effect of 80-90%. Thus, this effect was not dependent on VHL status.

Since the possibility exists that cyclopamine may affect other pathways we used an alternate approach to inhibit the SHH pathway using siRNA targeting key components of this pathway, i.e the Smo receptor and the Gli1 transcription factor. In transient transfection assays, both siRNAs decreased cell growth in a time (Additional file 1A) and concentration-dependent (Additional file 1B) manner by up to 80% at day 4. Such effects were observed in our panel of human CRCC cell lines (Additional file 1C) and again, this effect was mainly due to inhibition of cell proliferation, as assesed by BrdU incorporation (Additional file 1D).

Taken together, these data show that the inhibition of the SHH pathway decreases tumor cell growth essentially by affecting cell proliferation.

### SHH signaling pathway inhibition increases human CRCC cell apoptosis but not senescence

**Figure 4:**
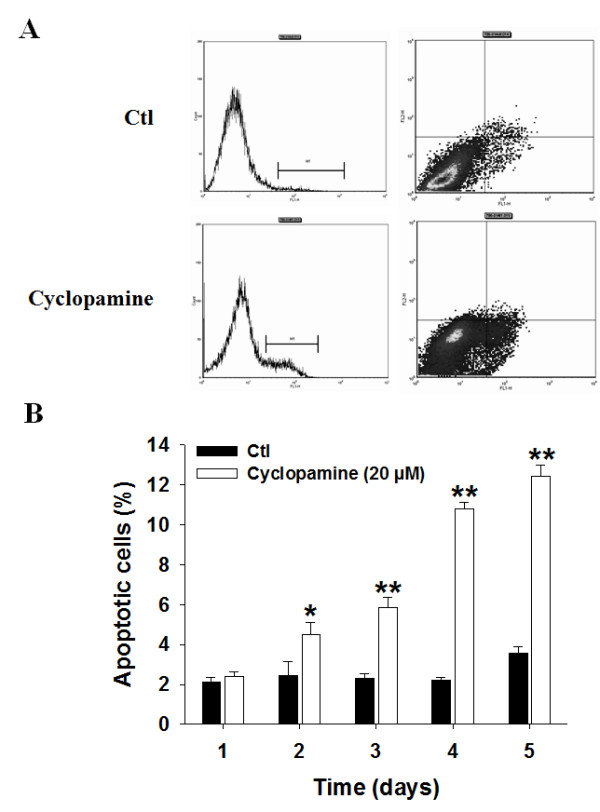
**The inhibition of the SHH signaling pathway induces human CRCC cells apoptosis**. FACS analysis of 786-0 treated in control (Ctl) or with cyclopamine (Cyclopamine) at 20 μM. (*A*) Exemples of FACS analysis (day 4). No evidence of necrosis was observed in any cases. (*B*) Quantitative analysis of apoptotic cells as a function of the time of treatments. Results are shown as mean ± SEM, n = 6 *, *P *< 0.05 and **, *P *< 0.01 from Ctl apoptosis. Bars, 5 μm.

Because the inhibition of cell proliferation by cyclopamine was not complete we also assessed whether the inhibitor was inducing apoptosis in human CRCC cells. Cyclopamine was inducing cell apoptosis in a time-dependent manner reaching a maximal induction of cell apoptosis of 12% (Figure 4A and 4B). As for cell proliferation assays, similar effects were observed in cells transiently transfected with siRNAs targeting Smo and Gli1 (Additional file 2). No effects of cyclopamine treatment were observed on tumor cell senescence (Additional file 3).

Thus, the growth inhibitory effects of SHH pathway inhibition is obtained mainly through a decrease of cell proliferation and in a lesser degree through induction of cell apoptosis in human CRCC.

### Transfection with Smo and Gli1 expression vectors alleviates the growth inhibitory effects of cyclopamine in human CRCC cells

To argument further the adequate targeting of cyclopamine against the SHH signaling pathway, we transiently transfected 786-0 cells for 0 to 5 days with Smo and Gli1 expression vectors (pCMV6-XL5-Smo and pCMV6-XL5-Gli1, respectively) or vector alone (pCMV6-XL5). We then assessed and compared the effects of cyclopamine on cell growth in cells transfected with these vectors and in untransfected cells. The overexpression of Smo and Gli1 was maximal 2 to 3 days post-transfection as assessed by western blot and quantitative RT-PCR (data not shown). The transfection with vector alone did not affect tumor cell proliferation at any time (Additional file 4). Interestingly, the transfection with Smo or Gli1 vector significantly increased cell proliferation 2 to 3 days post-transfection by up to 20-25% (Additional file 4). As expected from results presented on Figure 3, cyclopamine alone decreased cell proliferation by up to 80% at day 5 (Additional file 4). While the transfection with vector alone did not affect the inhibitory effect of cyclopamine on cell proliferation, the transfection with either Smo or Gli1 vectors alleviated significantly the growth inhibitory effect of cyclopamine at all times tested (Additional file 4).

These results show that overexpression of key components of the SHH signaling pathway not only has growth stimulatory effects on tumor cells but also alleviates the growth inhibitory effect of cyclopamine. These data clearly argument that the effect of cyclopamine is the consequence of SHH signaling pathway inhibition.

### Specificity of cyclopamine towards the SHH signaling pathway in human CRCC cells

**Figure 5:**
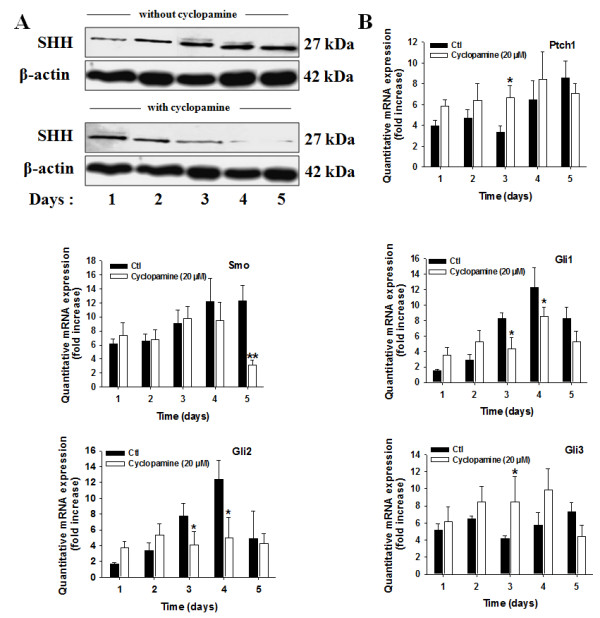
**Cyclopamine treatment decreases the expression of the SHH ligand, Smo receptor, Gli1 factors, and increases the expression of the Ptch1**. (*A*) Human 786-0 CRCC cells were seeded and treated for the indicated periods of time in control (Ctl) or with cyclopamine (Cyclopamine, 20 μM). Shown are western blot analysis of the SHH ligand in cell lysates from cells treated in each condition and incubated with antibodies against human SHH ligand and corresponding β-actin. The gels shown are representative for at least 3 independent experiments. (*B*) Human 786-0 CRCC cells were seeded and treated for the indicated periods of time in control (Ctl) or with cyclopamine (Cyclopamine, 20 μM). Quantitative gene expression of Ptch1, Smo receptors, Gli1, Gli2 and Gli3. Results are shown as mean ± SEM, n = 6 *, *P *< 0.05 from cells treated in Ctl at the same time point.

To check further the specificity of the inhibitor towards the SHH signaling pathway, we measured the expression of all the molecular components of the pathway by western blot or quantitative analysis of mRNAs expression in 786-0 cells. The expression of the SHH ligand was surprisingly, but interestingly, decreased as a function of time by cyclopamine, suggesting that the SHH ligand may itself be a target of the SHH pathway (Figure 5A).

Cyclopamine also decreased the expression of Ptch1 and, interestingly, of Smo receptors (Figure 5B), suggesting further that Smo may also be a target of the SHH pathway. Cyclopamine treatment decreased the expression of the transcription factors Gli1 and Gli2 (Figure 5B). The expression of Gli3, the endogenous repressor of the SHH pathway, was increased by cyclopamine treatment (Figure 5B). The effect of the inhibitor on gene expression was observed with different velocities from one component to another.

Overall, these results argue further for the specificity of the Smo inhibitor towards the SHH signaling pathway, and put in evidence two additional targets of the pathway, Ptch1 and Smo receptors.

### Cyclopamine injection induces tumor regression in nude mice bearing human CRCC tumors

**Figure 6:**
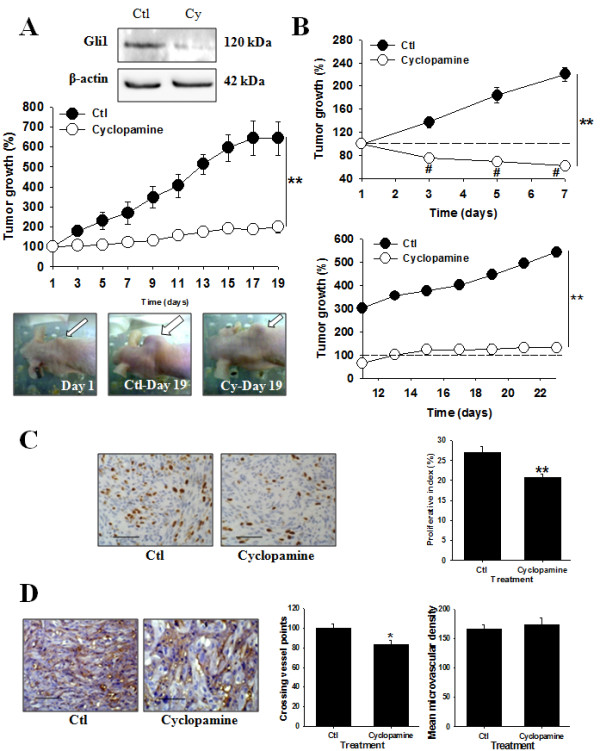
**Inhibition of the SHH signaling pathway induces tumor regression in nude mice**. (*A*) Tumor growth in mice treated according to the first experimental protocol. Results are shown as mean ± SEM, n = 7 for both groups; **, *P *< 0.01 cyclopamine-treated mice vs Ctl-treated mice. Immunoblotting experiments to measure expression of Gli1 in tumors lysates. The gels shown are representative for at least 3 independent experiments. Photographs show the implanted tumors in representative mice at day 1 of drug injection, at day 19 in Ctl-treated group or cyclopamine-treated group. (*B*) Tumor growth in mice treated according to the second protocol. Results are shown as mean ± SEM, n = 7 for both groups; **, *P *< 0.01 cyclopamine-treated mice vs Ctl-treated mice. (*C*) Left, tumor sections of control- (Ctl) or cyclopamine (Cyclopamine)-treated mice immunostained with an antibody against Ki67 (magnification ×400). Right, proliferative index. Results are shown as mean ± SEM, n = 7. **, P < 0.01 from Ctl-treated mice. (*D*) Left, tumor sections of control- (Ctl) or cyclopamine (Cyclopamine)-treated mice immunostained for CD31 (magnification ×400). Right, quantification of neovascularization. Results are shown as mean ± SEM, n = 7. *, P < 0.05 from Ctl-treated mice.

We next analyzed the effect of cyclopamine *in vivo *in the tumor xenografted nude mice model. In the first protocol (injections every other day), tumor growth was completely abolished by cyclopamine treatment (Figure 6A and Additional file 5A). The expression of Gli1 was decreased by 80% in tumors harvested from cyclopamine-treated mice compared to tumors from control mice showing adequate targeting of the drug (Figure 6A, top gels).

The anti-tumor effect obtained following the first protocol prompted us to assess in a second protocol whether we could observe tumor regression with cyclopamine by increasing the overall dose of the SHH inhibitor in tumor-bearing mice. In the second protocol (daily injections), cyclopamine induced more than 50% tumor regression (Figure 6B and Additional file 5B). The expression of Gli1 was also substantially decreased in tumors harvested from cyclopamine-treated mice by more than 80% (data not shown).

To assess wether the inhibitory effect on tumor growth of cyplopamine was long-lasting, in the mice treated using the second protocol, the control and cyclopamine treatments were stopped at day 10 and tumors were left growing for an additional 14 days period. In mice treated with cyclopamine, tumors did not grow further while in control mice the tumors' volume doubled (Figure 6B).

We used tumors harvested from mice treated according to the first protocol to assess the effect of cyclopamine on cell proliferation, death and on angiogenesis. Indeed for the second protocol mice were left untreated for several days and this not allow us to determine the effect of the drug on such tumor parameters. The proliferative index was significantly decreased by about 25% in mice treated with cyclopamine compared to mice treated in control (Figure 6C). Curiously, cyclopamine treatment did not influence tumor cell apoptosis (Additional file 5C). However such an effect may be due to the time between the last injection of cyclopamine and analysis, i.e 3 days. Very interestingly, tumor neovascularization was decreased significantly by cyclopamine treatment (Figure 6D).

These results suggest that the SHH signaling pathway plays a critical role in tumor growth *in vivo *mainly by affecting cell proliferation and vessel generations in human CRCC tumors.

### The SHH signaling pathway plays orchestral roles in oncogenic pathways stimulation in human CRCC

We next investigated the connection between the SHH signaling and known oncogenic pathways, i.e the PI3K/Akt (and GSK3), NF-κB and MAPK pathways. For that, we used cyclopamine or cells transiently transfected with siSmo or siGli1 targeting siRNAs alone or in combination with inhibitors of oncogenic pathways in 786-0 cells. The inhibitory effect of cyclopamine on cell growth was not additive with the effects of inhibitors of each pathway, suggesting strongly that the SHH signaling is linked to the activity of GSK-3 and to the oncogenic PI3K/Akt, NF-κB and MAPK pathways (Additional file 6). The effects of the GSK-3 and NF-κB inhibitors alone was observable only at day 1 and day 2 of treatments, while the effect of the PI3K/Akt and MAPK inhibitors lasted during the 5 days of the experiments (Additional file 6), suggesting a sequential activation of these pathways. Similar results were obtained after Smo or Gli1 silencing (Additional file 7).

**Figure 7:**
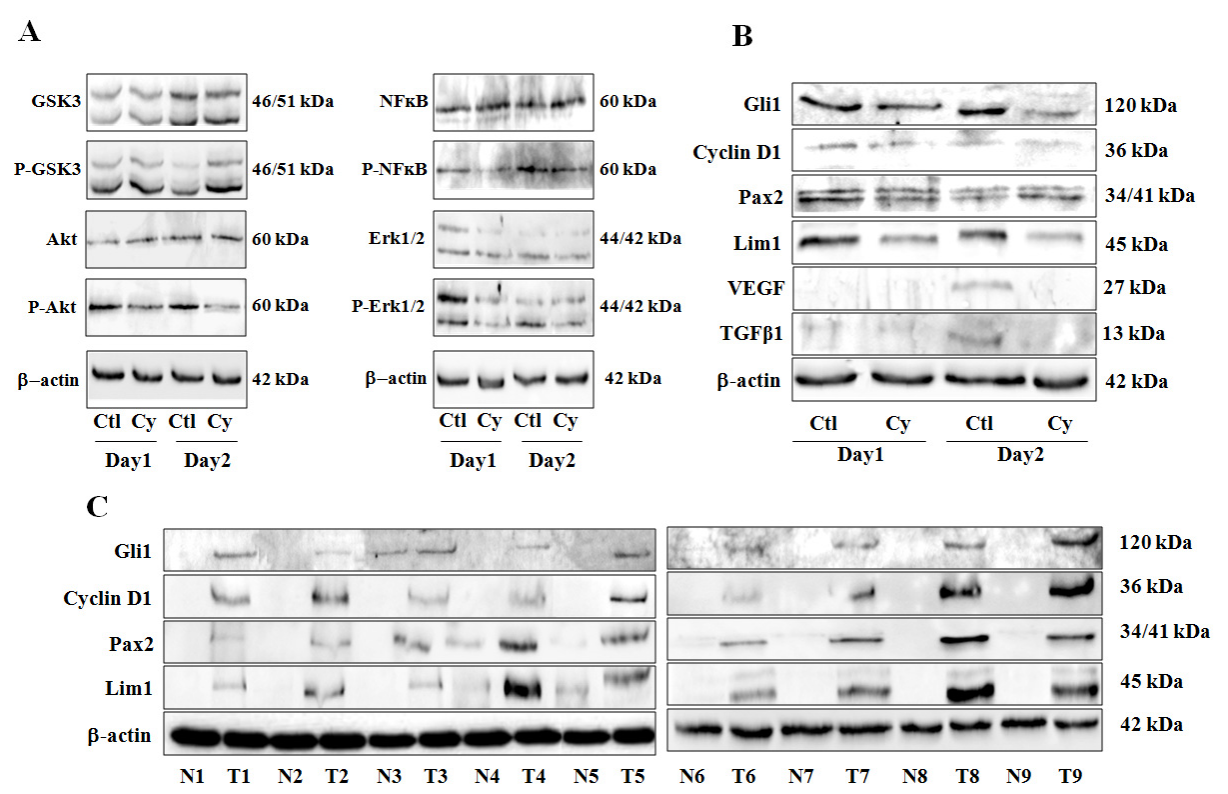
**The SHH signaling pathway plays a pivotal and orchestral role in the constitutive activation of oncogenic pathways in human CRCC**. (*A*) Western blots analysis of human CRCC 786-0 cell lysates treated for 2 days in control (Ctl) or with cyclopamine (Cy) at 20 μM and incubated with the antibodies against non-phosphorylated GSK-3 (GSK-3), phospho-GSK-3 (P-GSK3), non-phosphorylated Akt (Akt), phospho-Akt (P-Akt), non-phosphorylated NF-κB (NF-κB), phospho-NF-κB (P-NF-κB), non-phosphorylated Erk1/2 (Erk1/2), phospho-Erk1/2 (P-Erk1/2) and corresponding β-actin. The gels shown are representative for at least 3 independent experiments. (*B*) Western blots analysis of human CRCC 786-0 cells lysates treated for 2 days in control (Ctl) or with cyclopamine (Cy) at 20 μM and incubated with the antibodies against Gli1, cyclin D1, Pax2, Lim1, VEGF, TGF-β1 and corresponding β-actin. The gels shown are representative for at least 3 independent experiments. (*C*) Western blot analysis in 9 human tumors (T1 ...) and normal corresponding tissues (N1 ...) lysates incubated with antibodies against Gli1, cyclin D1, Pax2, Lim1, and corresponding β-actin. The gels shown are representative for at least 3 independent experiments.

We next evaluated the effect of cyclopamine and of Smo and Gli1 silencing through transient transfection on GSK-3 activation and of all of the above-mentioned signaling pathways by western blot in 786-0 cells. The non-phosphorylated states of GSK-3, Akt, NF-κB and Erk1/2 remain unchanged after cyclopamine treatments (Figure 7A). However, cyclopamine treatments induced a decrease in the phosphorylation state of Akt, NF-κB and Erk1/2, and an increase in the phosphorylated state of GSK-3 (Figure 7A), thus inhibiting their biological activities. Again, similar results were obtained after Smo or Gli1 silencing (Additional file 8).

These results argue for an orchestral role for SHH signaling in the constitutive activation of oncogenic pathways in this pathology.

We tested a panel of genes known for some of them to be Gli's targets in other cell lines or tissue types and shown to be important in human CRCC tumorigenesis, i.e Gli1 itself, cyclin D1, Pax2, Lim1, VEGF and TGF-β. By treating 786-0 cells with cyclopamine for 1 or 2 days, we showed that all of the tested targets were under the transcriptional activity of the SHH signaling pathways except cyclin D1, and that Pax2 expression was only inhibited at day 1 of cyclopamine treatment (Figure 7B).

In all patients tested, Gli1, cyclin D1, Pax2 and Lim1 were expressed exclusively in tumors at all stages (Figure 7C). The expression of VEGF and TGF-β were not assessed in these patients because these factors are known to be expressed in tumors and in a lesser degree in normal counterparts in human CRCC [2].

In conclusion, various Gli target genes have found to be specifically expressed in tumors, clearly argumenting the pivotal role played by the SHH signaling pathway in human CRCC.

## Discussion

The SHH signaling pathway plays crucial roles in metazoan embryo patterning [16]. During nephrogenesis, the biological effects of the SHH signaling pathway concern cell differentiation, migration and growth as well as angiogenesis [17]. Inherited or acquired modifications or abberations in components of the SHH cascade result in various phenotypes such as congenital anomalies (Pallister-Hall syndrome and holoprosencephaly) and various cancers including basal cell carcinoma and gastrointestinal cancers [18,19].

We show that this pathway is constitutively expressed and activated in human CRCC both *in vitro *and *in vivo *in freshy harvested tumors and in tumors grown in nude mice. The SHH ligand was expressed in cells and tumors but there was no consensus as for a preferential expression in tumors vs. normal corresponding tissues. This may be explained in part by diffusion of the SHH ligand secreted by the tumor to the adjacent normal tissues. Alternatively, some cells, such as resident stem cells, may expressed SHH ligand as suggested by other studies, arguing for a role for SHH pathway in the maintenance of the stem cell compartment [20,21]. Our results clearly show that the SHH signaling pathway is active in tumors but not in normal kidney tissues, as evidenced by the elevated expression of Smo and Gli transcription factors in tumors vs. corresponding normal tissues. As no data has been reported about the involvement of the SHH signaling pathway in human CRCC, it remains unknown whether there are activating mutations of this pathway. Our data suggest that the erroneous activation of this pathway in human CRCC may results from the expression of the Ptch1 receptor and the signaling components Smo and Gli.

The SHH ligand was present in all cell lines tested whether or not they are expressing VHL and the level of expression of SHH, Smo, Gli1, Gli2 and Gli3 were identical in 786-0 cells untransfected or VHL constructs-transfected cells. Although some studies have reported crosstalk between SHH and HIF pathways in other systems [22], our data suggest that the activation state of the SHH signaling is not associated with the VHL/HIF system in human CRCC.

Our results show that the SHH signaling pathway promotes tumor cell growth in human CRCC, regardless of the VHL status. The specificity of the Smo inhibitor cyclopamine against the SHH signaling pathway was clearly demonstrated herein by showing that overexpression of Smo and Gli1 alleviates the growth inhibitory effect of cyclopamine and by the negative effect of the Smo inhibitor on the expression not only of the SHH ligand but also of Gli1 and Gli2. Surprisingly, the expression of Ptch1 was increased by cyclopamine treatment, suggesting that Ptch1 expression might be repressed by the transcriptional activity of the SHH signaling pathway in human CRCC; this contrasts with what has been observed in other systems [15]. The expression of Smo was also decreased by the Smo inhibitor but at later time points suggesting that Smo may be transcriptionnally regulated by Gli transcription factors. In human CRCC, we show, using various experimental approaches, i.e cyclopamine, Smo and Gli1 targeting siRNAs and Smo and Gli1 overexpression, that the SHH signaling pathway stimulates essentially cell proliferation and in a lesser degree inhibits cell death, and no effects were observed on tumor cell senescence.

Interestingly, SHH signaling inhibition induced substantial tumor regression in nude mice, and the inhibitory effect on tumor growth was long-lasting after treatment arrest. Such spectacular effects of SHH signaling inhibition on tumor growth were also observed in other cancers such as human cholangiocarcinoma and melanomas [23]. Herein, we also showed that the treatment of human CRCC tumor-bearing nude mice with cyclopamine decreases tumor vascularization, indicating that the SHH pathway stimulates neoangiogenesis in human CRCC. Moreover, we showed that the expression of the angiogenic and growth factors VEGF and TGF-β are under the transcriptional control of the SHH signaling pathway, and thus that they are probably part of the targets mediating this effect in human CRCC. However, reports of the prognostic value of vascularization in human CRCC have shown either no effect on patient survival, better survival or worse prognosis [24-26]; these discrepancies may be the consequence of vessel size and/or the co-existence of different vessels depending on the expressed markers CD31 and CD34 [27].

The PI3K/Akt, NF-κB, MAPK, Jun kinase, Notch and SHH signaling pathways have been shown to be the main signaling events involved in nephrogenesis [28,29]. Interestingly, these pathways are activated constitutively in human CRCC. Our results demonstrate clear interactions between the PI3K/Akt, NF-κB, MAPK, and SHH signaling pathways in human CRCC. As GSK-3 has been shown to inhibit Glis functions [30], it was surprising to observe that GSK-3 phosphorylation was increased in response to SHH inhibition using cyclopamine and Smo and Gli1 targeting siRNAs. However, the Akt-independent phosphorylation of GSK-3 may have opposite effect on GSK-3 activity. Finally, NF-κB has been shown to contribute to SHH signaling activation through SHH ligand induction in pancreatic cells [31]. The inhibitory effect of cyclopamine and of Smo and Gli1 silencing on NF-κB activation observed here thus suggests that the SHH signaling stimulates NF-κB, which itself stimulates SHH signaling. Therefore, our results provide evidence for a pivotal and orchestral role for SHH signaling pathway in the constitutive activation of oncogenic pathways leading to sustained tumor growth.

As stated above, various Gli targets have been evidenced [15]. We identified various genes being under the transcriptional activity of Gli. There are some reports in the literature describing the involvement of cyclin D1 and Pax2 in human CRCC tumorigenesis [32,33] and for Pax2 in responses to therapies, but not for the SHH ligand, Gli1 and Lim1. Interestingly, the SHH ligand itself was shown to be a transcriptional target of the SHH signaling. Thus, the system boosts itself by also increasing the expression of the ligand.

## Conclusions

Until the recent development of targeted therapies with multi-tyrosine kinase receptors inhibitors such as sunitinib and sorafenib, and although their effects are not long-lasting due to therapy-induced resistance, there was no efficient treatment for advanced human CRCC. Our results indicate that inhibition of SHH signaling might represent a new and complementary therapeutic approach against human CRCC. As SHH signaling pathway has emerged as a crucial pathway in the pathogenesis of various tumor types, SHH inhibitors are currently being evaluated as potential anticancer drugs. Here, we showed that cyclopamine was safe and well tolerated by the mice, providing the proof of concept for the use of this family of drugs *in vivo*.

Overall, we showed that the SHH pathway is specifically reactivated in human CRCC and that targeting this pathway might be particularly efficient against this disease, not only through inhibition of tumor growth but also by impeding tumor vascularization. Because CRCC is resistant to therapies, describing and understanding all the molecular mechanisms leading to carcinogenesis is critical to develop treatment for this cancer type. Thus, our study identifies the SHH pathway as an important signaling pathway implicated in kidney tumorigenesis.

## Methods

### Cell culture and reagents

Human CRCC cell lines either deficient in VHL (786-0, UOK-126, A498) or expressing VHL (ACHN, Caki-2) as described [9]. Clones of 786-0 cells transfected either with human VHL gene (786-0 VHL), inactive troncated human VHL gene (786-0 ΔVHL), or the vector alone only pCR3-Uni (786-0 V) were also used.

### Human tumor biopsies

The tumor and normal corresponding tissue of 9 patients were obtained in collaboration with the Department of Urology (Pr. D. Jaqmin and Pr. H. Lang) of the "Nouvel Hôpital Civil" (NHC), Strasbourg, France. Informed consent was obtained from all patients. The tumors were staged according to the tumor node metastasis (TNM) classification [34]: 2 pT1aNx, 1 pT1bNx,, 1 pT2N0, 1 pT2Nx,, 1 pT3aNx, 2 pT3bN0 and 1 pT3bN1 (T1 to T9). Immediately after surgical resection, tissues were fresh frozen and kept in liquid nitrogen until RNA and protein expression analysis.

### Western Blot Analysis

Protein extractions and membrane preparations were performed as described [35]. Membranes were incubated overnight at 4°C with the appropriate dilution of the following primary antibodies: anti-Akt antibody (1:250; Millipore), anti-phospho-Akt antibody (1:150; Ozyme, Cell signaling local distributor, Saint-Quentin-en-Yvelines, France), anti-GSK3_α/β _antibody (1:1000; Millipore 05-903), anti-phospho-GSK3_α/β _(Ser21/9) antibody (1:250; Ozyme), anti-NF-κB (1:2000; Millipore AB1604), anti-phospho-NF-κB (S468) (1:250; Ozyme); anti-Erk1/(1:1000; Ozyme), anti-phospho-Erk1/2 (1:1000; Millipore 05-481), anti-SHH (1:500; Ozyme), anti-cyclinD1 (1:750; Ozyme), anti-Gli1 (1:2000; Millipore AB3444), anti-Pax2 (1:1000; Ozyme), anti-Lim1 (1:3000; Millipore AB3200), anti-VEGF (1:250; Millipore MAB3734) and anti-TGFβ1 (1:200; Ozyme). For visualization of protein gel loading, an anti β-actin antibody was used (1:5000; Sigma-aldrich, St Quentin Fallavier, France). The appropriate horseradish peroxidase-conjugated secondary was used. Immunoreactivity was visualized as detailed [35].

### Real-time quantitative RT-PCR analysis

Total RNAs were extracted from CRCC cells and tissues using the Trizol method according to the manufacturer's protocol (Invitrogen). Five μg of total RNA were reverse transcribed in a reaction buffer (Invitrogen) and non-specific primer p(dT)_15 _(Roche Diagnostics, Meylan, France), at 37°C for 1 h. cDNAs specific for each Ptch1, Smo, Gli1, Gli2, Gli3 and SHH mRNAs were amplified using the "LightCycler-FastStart DNA Master SYBR (syber) Green" kit (Roche Diagnostics). Sense and antisens primers used are depicted in Additional file 9. Each sample was analyzed 3 times and quantified with the analysis software for LightCycler (Roche Diagnostics).

### Cell density

CRCC cell proliferation was assessed by counting adherent cells, as described [35]. RCC cells were seeded in 24-well plates (20,000 cells/ml), grown for 24 h, and then treated for 1-5 days with various concentrations of cyclopamine (LC Laboratories, Woburn, USA), SB216763 (GSK3 inhibitor, Sigma-Aldrich), LY294002 (PI3K inhibitor, Sigma-Aldrich), BAY 11-7085 (NF-κB inhibitor, Calbiochem, Fontenay-sous-Bois, France), or U0126 (MAPK inhibitor, Calbiochem), alone or in combination, as indicated in the appropriate Figures or Figure legends, or the diluent only (DMSO). In some experiments, we also used Smo and Gli1 targeting siRNAs and Smo and Gli1 expressing vector and assessed cell density, either alone or in combination with cyclopamine or the above-mentioned oncogenic pathways inhibitors, as indicated in the appropriate Figures or Figure legends.

### Bromodeoxyuridine (BrdU) incorporation

CRCC cells were seeded in 96-well plate (20,000 cells/ml), grown for 24 h and FBS was replaced by 0,1% of BSA during an additional 24 h to render cells quiescent. Cells were treated for 1-5 days with 20 μM cyclopamine or the corresponding volume of DMSO. In some experiments, we also used Smo and Gli1targeting siRNAs and performed BrdU incorporation studies, as indicated in the appropriate Figures or Figure legends. Test was then realized according to the protocol of the manufacturer (Calbiochem^®^, Merck KGaA, Darmstadt, Germany).

### Fluorescence-Activated Cell Sorting Analysis

CRCC cells were seeded in 6-well plates (20000 cells/ml) and treated with 20 μM cyclopamine or DMSO. In some experiments, we also used Smo and Gli1targeting siRNAs and performed fluorescence-activated cell sorting (FACS), as indicated in the appropriate Figures or Figure legends. Floating and adherent cells were harvested and resuspended in incubation buffer (100 μL/500 000 cells: 140 mmol/L NaCl, 5 mmol/L CaCl_2_, and 10 mmol/L HEPES buffer) containing Annexin V-FITC and propidium iodide (1 μg/mL) and incubated in a dark chamber at 4°C for 10 minutes. After centrifugation, the supernatant was withdrawn and cells fixed in a dark chamber in 200 μL of formol 1% at 4°C for 10 min. After centrifugation, cells were resuspended in 200 μL incubation buffer and subjected to FACS analysis. Fluorescence analysis were performed using FACSort flow cytometer (BD) and the fraction of viable cells, and apoptosis cells was determined using FCS express software (DeNovo Software, Los Angeles, CA).

### Xenograft Tumor Model

All animal studies were in compliance with the French animal use regulations. Four million 786-0 cells were injected s.c. under the skin of 4 week-old athymic male mice (SWISS nu-/nu-; Charles River Laboratories, l'Arbresle, France). Tumor volumes were measured as previously described [35]. We begun drug injections when 786-0 tumors had grown to an overall volume of 100 mm^3^. We followed two protocols: the first protocol was injection of cyclopamine i.p at 0.5 mg/mouse at 2 days interval for 19 days and the second protocol was injection of cyclopamine i.p at 0.4 mg/mouse every day for 7 days, the control groups receiving the vehicle alone (DMSO/oil, v/v) at the same time period. Mice were thus divided in 4 groups, two groups treated with cyclopamine and 2 groups treated in control, according to the 2 protocols. For the second protocol, the treatment was then followed for 4 days (day 7 to day 11) and mice were then left untreated for additional 12 days (day 11 to day 23), and tumors growth was measured. At the end of the treatments, animals were sacrified and the tumors were harvested, paraffin embedded, and cut in 4-μm-thick sections for subsequent immunohistochemical analysis as described before for the proliferative index, the apoptotic index and the neovascularization and snap-frozen for PCR or Western blot analysis.

### Statistical analysis

All values are expressed as mean ± s.e.m. Values were compared using multifactorial analysis of variance followed by the Student-Newman-Keul's test for multiple comparisons. A *P *< 0.05 was considered significant.

## Abbreviations

CRCC: clear cell renal cell carcinoma; Ptch: patched1 receptor; Smo: smoothened receptor; VHL: von Hippel-Lindau.

## Competing interests

The authors declare that they have no competing interests.

## Authors' contributions

VD, TM designed research. VD, SD, LT, SR performed research. JJH, DJ, HL contributed clinical material. VD, CC, VL, TM analyzed data. TM wrote the paper. All authors read and approved the manuscript.

## Supplementary Material

Additional file 1 **The silencing of the SHH signaling pathway decreases human CRCC cell proliferation**. Tumor cells were seeded in 24-well plates (20,000 cells/ml), grown for 24 h and were then transiently transfected for 24 to 96 h with Smo-targeting siRNA (siSmo), Gli1-targeting siRNA (siGli1) or control siRNA (siCtl), according to the manufacturer's instructions (Applied Biosystems, Ambion local distributor, Courtaboeuf, France). (A) Human 786-0 cells were transiently transfected with siRNA (siCtl, siSmo and siGli1, as indicated) at 100 nM or not transfected (Ctl) and cells were counted each day. Results are shown as mean ± SEM, n = 6; *, *P *< 0.05; **, *P *< 0.01 from Ctl. (B) Human 786-0 cells were transiently transfected for 4 days with siRNA (siCtl, siSmo, and siGli1, as indicated) or not transfected (Ctl) at the concentrations indicated in the figure and adherent cells were counted. Results are shown as mean ± SEM, n = 6; **, *P *< 0.01 from Ctl. (C) Our panel of human CRCC cells either deficient or expressing the VHL gene were transiently transfected for 4 days with siRNA (siCtl, siSmo, and siGli1, as indicated) at 100 nM or not transfected (Ctl) and adherent cells were counted. Results are shown as mean ± SEM, n = 6; **, *P *< 0.01 from Ctl that was set to 100%. (D) Our panel of human CRCC cells either deficient or expressing VHL were analyzed for BrdU incorporation after transient transfection with siRNA (siCtl, siSmo, and siGli1, as indicated) at 100 nM or not transfected (Ctl) for the indicated periods of time. For clarity, only the results concerning 786-0 cell line are presented since similar results were obtained with the other cell lines (dat not shown). Results are shown as mean ± SEM, n = 6; *, P < 0.05; **, *P *< 0.01 from corresponding Ctl. Click here for file

Additional file 2 **The silencing of the SHH signaling pathway induces tumor cells apoptosis**. FACS analysis of 786-0 cells transiently transfected with siRNA (siCtl, siSmo and siGli1, as indicated) at 100 nM or not transfected (Ctl). No evidence of necrosis was observed in any cases. The percent of apoptotic cells was quantified as a function of treatment times. Results are shown as mean ± SD, n = 6 *, *P *< 0.05 and **, *P *< 0.01 from Ctl apoptosis. Click here for file

Additional file 3 **The inhibition of the SHH signalling pathway does not induce CRCC cells senescence**. RCC cells were seeded in 24-well plates (20,000 cells/ml), grown for 24 h, and then treated for 1 day to 5 days with 20 μM cyclopamine or the corresponding volume of DMSO. The test was then realized according to the protocol of the manufacturer ("Senescence β-Galactosidase Saining Kit", Cell Signaling). β-galactosidase staining was analysed under a microscope (magnification × 200) (A) Exemples of microscope analysis (day 4). (B) Quantitative analysis of senescent cells as function of the time of treatments, the number of total and stained cells in 8 fields (0,25 cm2 each) were quantified in a blinded manner to determine the percentage of senescent cells. Results are shown as mean ± SEM, n = 6 from Ctl senescence. Bars, 5 μm. Click here for file

Additional file 4 **The overexpression of Smo and Gli1 alleviate the growth inhibitory effect of cyclopamine in human tumor cells**. Human 786-O cells were seeded in 24-well plates (20,000 cells/ml), grown for 24 h and were then treated with cyclopamine (20 μM) or transiently transfected for 1 to 5 days with cDNA overexpression plasmids (pCMV6-XL5 vector, pCMV6-XL5-Smo and pCMV6-XL5-Gli1) according to the manufacturer's instructions (Clinisciences, Origene local distributor, Montrouge, France), either alone or in combination with cyclopamine, as indicated in the figure, and adherent cells were counted each day. Results are shown as mean ± SEM, n = 6; **, *P *< 0.01 from Ctl; #, P < 0.01 from cyclopamine alone or in cells transfected with vector alone at 72 h, 96 h and 120 h. Click here for file

Additional file 5 **Inhibition of the SHH signaling pathway induces tumor regression in nude mice**. (A) Tumor weight in mice treated according to the first experimental protocol. Results are shown as mean ± SEM, n = 7 for both groups; **, *P *< 0.01 cyclopamine-treated mice vs Ctl-treated mice. (B) Tumor weight in mice treated according to the second experimental protocol. Results are shown as mean ± SEM, n = 7 for both groups; **, *P *< 0.01 cyclopamine-treated mice vs Ctl-treated mice. (C) Left, tumor sections of control- (Ctl) or cyclopamine (Cyclopamine)-treated mice immunostained for DNA fragmentation (magnification ×400). Right, apoptotic index. Results are shown as mean ± SEM, n = 7; NS. Click here for file

Additional file 6 **The SHH signaling pathway plays a pivotal and orchestral role in the constitutive activation of oncogenic pathways in human CRCC**. Human CRCC 786-0 cells were seeded and treated for the indicated period of times in control (Ctl) or with cyclopamine (Cyclopamine) at 20 μM, the GSK-3 inhibitor SB216763 at 20 μM, the PI3K/Akt inhibitor LY294002 at 10 μM, the NF-κB inhibitor BAY 11-7085 at 2,5 μM or the MAPK inhibitor U0126 at 30 μM, either alone or in combination, as indicated in the figure, and adherent cells were counted each day. Results are shown as mean ± SEM, n = 6; **, *P *< 0.01 from Ctl. Click here for file

Additional file 7 **The silencing of the SHH signaling pathway further arguments the orchestral role of this pathway in oncogenic pathways activation in human CRCC**. Human CRCC 786-0 cells were seeded and transiently transfected with siRNA (siCtl, siSmo and siGli1) at 100 nM or not transfected (Ctl) and treated, for the indicated period of times, with the GSK-3 inhibitor SB216763 (SB) at 20 μM, the PI3K/Akt inhibitor LY294002 at 10 μM (LY), the NF-κB inhibitor BAY 11-7085 (BAY) at 2.5 μM or the MAPK inhibitor U0126 (U0) at 30 μM, either alone or in combination, as indicated in the figure, and adherent cells were counted each day. Results are shown as mean ± SEM, n = 6; **, *P *< 0.01 from Ctl. Click here for file

Additional file 8 **The SHH signaling pathway plays an orchestral role in the constitutive activation of oncogenic pathways in human CRCC as evaluated after Smo and Gli1 silencing**. Western blots analysis of human 786-0 cell lysates that were transiently transfected for 2 days with siRNA (siCtl, siSmo and siGli1, as indicated) at 100 nM and incubated with the antibodies against non-phosphorylated GSK-3 (GSK-3), phospho-GSK-3 (P-GSK3), non-phosphorylated Akt (Akt), phospho-Akt (P-Akt), non-phosphorylated NF-κB (NF-κB), phospho-NF-κB (P-NF-κB), non-phosphorylated Erk1/2 (Erk1/2), phospho-Erk1/2 (P-Erk1/2) and corresponding β-actin, as indicated. The gels shown are representative of at least 3 independent experiments. Click here for file

Additional file 9 **Primer pairs for the quantitative measurements of gene expression**. Table showing primer pairs for the quantitative measurements of gene expression. Click here for file
