# Supplementary figures and images for: The sonic hedgehog signaling pathway is reactivated in human renal cell carcinoma and plays orchestral role in tumor growth
Source: Mol Cancer. 2009 Dec 16;8:123. doi: 10.1186/1476-4598-8-123 (PMC2803450; doi:10.1186/1476-4598-8-123)

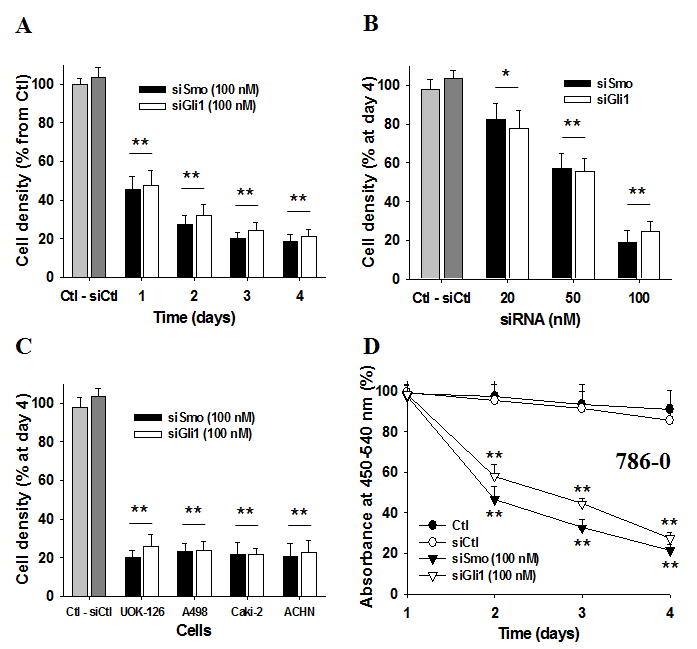

Supplement: Additional file 1 — The silencing of the SHH signaling pathway decreases human CRCC cell proliferation. Tumor cells were seeded in 24-well plates (20,000 cells/ml), grown for 24 h and were then transiently transfected for 24 to 96 h with Smo-targeting siRNA (siSmo), Gli1-targeting siRNA (siGli1) or control siRNA (siCtl), according to the manufacturer's instructions (Applied Biosystems, Ambion local distributor, Courtaboeuf, France). (A) Human 786-0 cells were transiently transfected with siRNA (siCtl, siSmo and siGli1, as indicated) at 100 nM or not transfected (Ctl) and cells were counted each day. Results are shown as mean ± SEM, n = 6; *, P < 0.05; **, P < 0.01 from Ctl. (B) Human 786-0 cells were transiently transfected for 4 days with siRNA (siCtl, siSmo, and siGli1, as indicated) or not transfected (Ctl) at the concentrations indicated in the figure and adherent cells were counted. Results are shown as mean ± SEM, n = 6; **, P < 0.01 from Ctl. (C) Our panel of human CRCC cells either deficient or expressing the VHL gene were transiently transfected for 4 days with siRNA (siCtl, siSmo, and siGli1, as indicated) at 100 nM or not transfected (Ctl) and adherent cells were counted. Results are shown as mean ± SEM, n = 6; **, P < 0.01 from Ctl that was set to 100%. (D) Our panel of human CRCC cells either deficient or expressing VHL were analyzed for BrdU incorporation after transient transfection with siRNA (siCtl, siSmo, and siGli1, as indicated) at 100 nM or not transfected (Ctl) for the indicated periods of time. For clarity, only the results concerning 786-0 cell line are presented since similar results were obtained with the other cell lines (dat not shown). Results are shown as mean ± SEM, n = 6; *, P < 0.05; **, P < 0.01 from corresponding Ctl. [file 1476-4598-8-123-S1.TIFF]

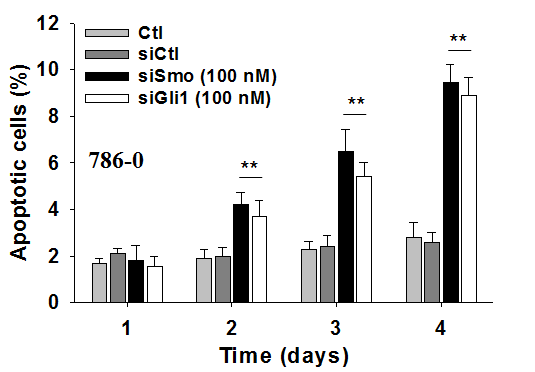

Supplement: Additional file 2 — The silencing of the SHH signaling pathway induces tumor cells apoptosis. FACS analysis of 786-0 cells transiently transfected with siRNA (siCtl, siSmo and siGli1, as indicated) at 100 nM or not transfected (Ctl). No evidence of necrosis was observed in any cases. The percent of apoptotic cells was quantified as a function of treatment times. Results are shown as mean ± SD, n = 6 *, P < 0.05 and **, P < 0.01 from Ctl apoptosis. [file 1476-4598-8-123-S2.TIFF]

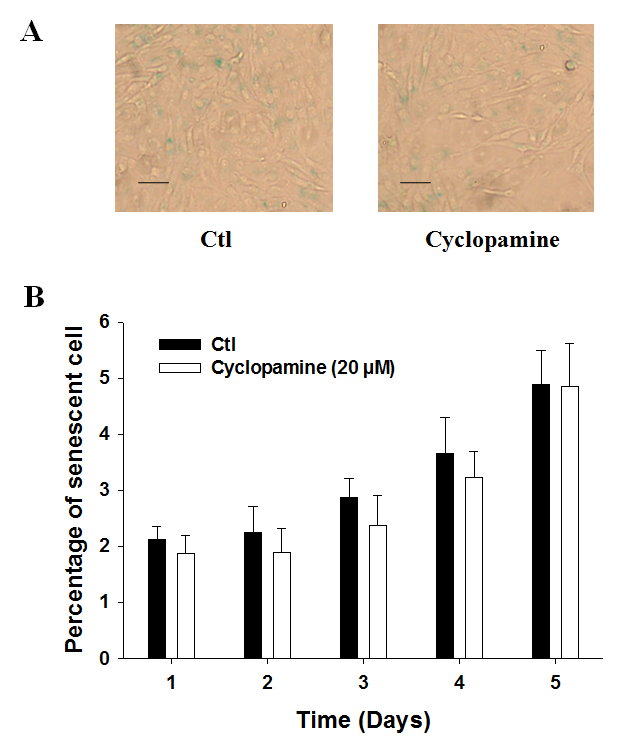

Supplement: Additional file 3 — The inhibition of the SHH signalling pathway does not induce CRCC cells senescence. RCC cells were seeded in 24-well plates (20,000 cells/ml), grown for 24 h, and then treated for 1 day to 5 days with 20 μM cyclopamine or the corresponding volume of DMSO. The test was then realized according to the protocol of the manufacturer ("Senescence β-Galactosidase Saining Kit", Cell Signaling). β-galactosidase staining was analysed under a microscope (magnification × 200) (A) Exemples of microscope analysis (day 4). (B) Quantitative analysis of senescent cells as function of the time of treatments, the number of total and stained cells in 8 fields (0,25 cm2 each) were quantified in a blinded manner to determine the percentage of senescent cells. Results are shown as mean ± SEM, n = 6 from Ctl senescence. Bars, 5 μm. [file 1476-4598-8-123-S3.TIFF]

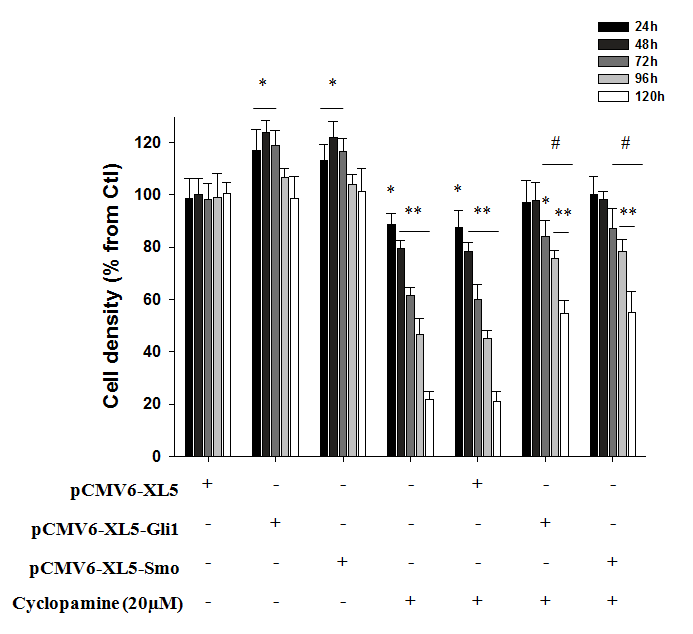

Supplement: Additional file 4 — The overexpression of Smo and Gli1 alleviate the growth inhibitory effect of cyclopamine in human tumor cells. Human 786-O cells were seeded in 24-well plates (20,000 cells/ml), grown for 24 h and were then treated with cyclopamine (20 μM) or transiently transfected for 1 to 5 days with cDNA overexpression plasmids (pCMV6-XL5 vector, pCMV6-XL5-Smo and pCMV6-XL5-Gli1) according to the manufacturer's instructions (Clinisciences, Origene local distributor, Montrouge, France), either alone or in combination with cyclopamine, as indicated in the figure, and adherent cells were counted each day. Results are shown as mean ± SEM, n = 6; **, P < 0.01 from Ctl; #, P < 0.01 from cyclopamine alone or in cells transfected with vector alone at 72 h, 96 h and 120 h. [file 1476-4598-8-123-S4.TIFF]

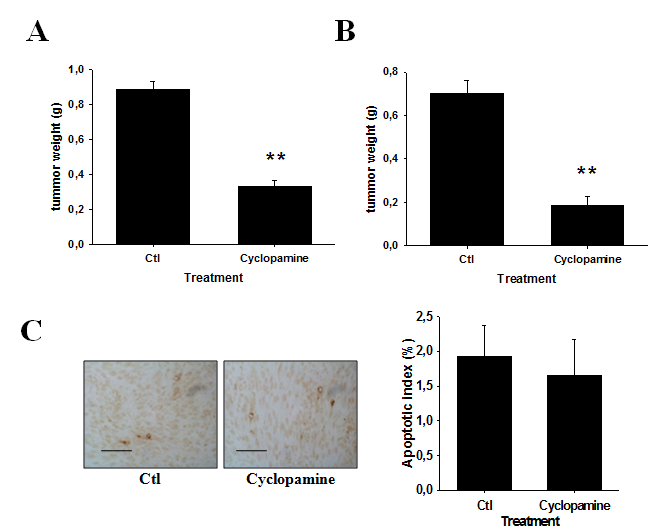

Supplement: Additional file 5 — Inhibition of the SHH signaling pathway induces tumor regression in nude mice. (A) Tumor weight in mice treated according to the first experimental protocol. Results are shown as mean ± SEM, n = 7 for both groups; **, P < 0.01 cyclopamine-treated mice vs Ctl-treated mice. (B) Tumor weight in mice treated according to the second experimental protocol. Results are shown as mean ± SEM, n = 7 for both groups; **, P < 0.01 cyclopamine-treated mice vs Ctl-treated mice. (C) Left, tumor sections of control- (Ctl) or cyclopamine (Cyclopamine)-treated mice immunostained for DNA fragmentation (magnification ×400). Right, apoptotic index. Results are shown as mean ± SEM, n = 7; NS. [file 1476-4598-8-123-S5.TIFF]

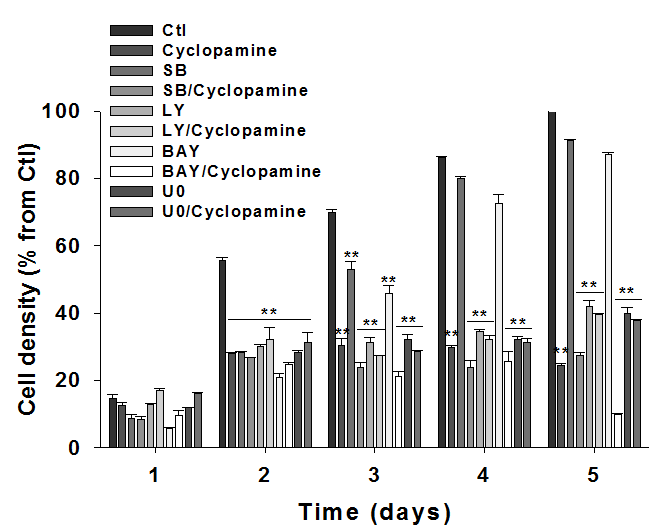

Supplement: Additional file 6 — The SHH signaling pathway plays a pivotal and orchestral role in the constitutive activation of oncogenic pathways in human CRCC. Human CRCC 786-0 cells were seeded and treated for the indicated period of times in control (Ctl) or with cyclopamine (Cyclopamine) at 20 μM, the GSK-3 inhibitor SB216763 at 20 μM, the PI3K/Akt inhibitor LY294002 at 10 μM, the NF-κB inhibitor BAY 11-7085 at 2,5 μM or the MAPK inhibitor U0126 at 30 μM, either alone or in combination, as indicated in the figure, and adherent cells were counted each day. Results are shown as mean ± SEM, n = 6; **, P < 0.01 from Ctl. [file 1476-4598-8-123-S6.TIFF]

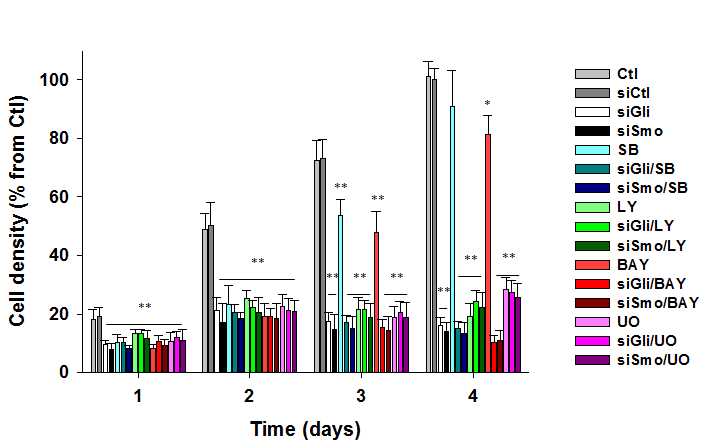

Supplement: Additional file 7 — The silencing of the SHH signaling pathway further arguments the orchestral role of this pathway in oncogenic pathways activation in human CRCC. Human CRCC 786-0 cells were seeded and transiently transfected with siRNA (siCtl, siSmo and siGli1) at 100 nM or not transfected (Ctl) and treated, for the indicated period of times, with the GSK-3 inhibitor SB216763 (SB) at 20 μM, the PI3K/Akt inhibitor LY294002 at 10 μM (LY), the NF-κB inhibitor BAY 11-7085 (BAY) at 2.5 μM or the MAPK inhibitor U0126 (U0) at 30 μM, either alone or in combination, as indicated in the figure, and adherent cells were counted each day. Results are shown as mean ± SEM, n = 6; **, P < 0.01 from Ctl. [file 1476-4598-8-123-S7.TIFF]

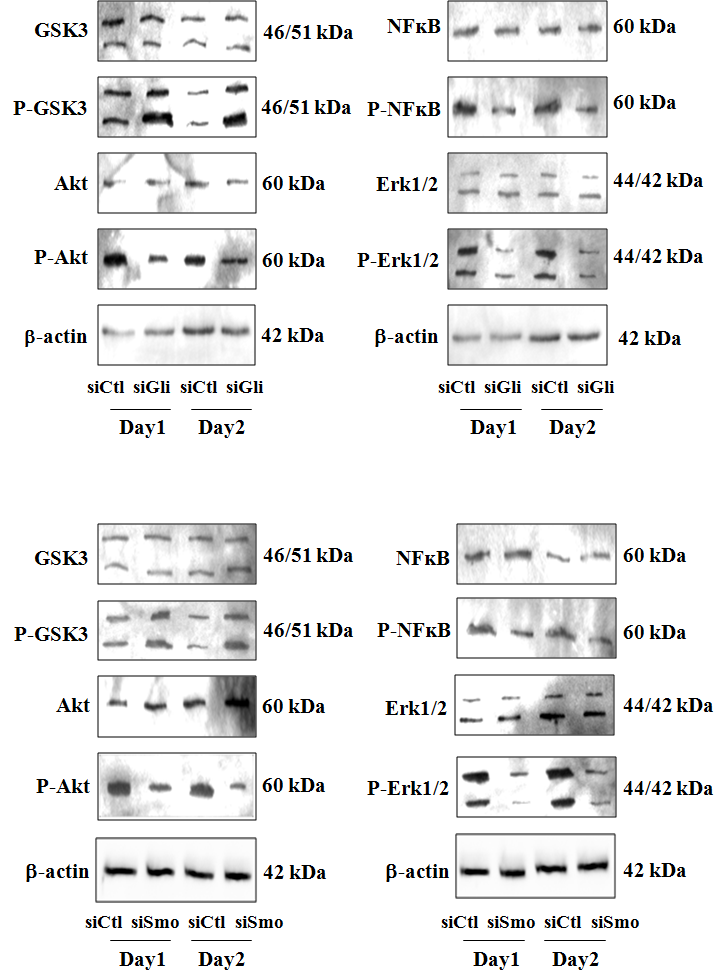

Supplement: Additional file 8 — The SHH signaling pathway plays an orchestral role in the constitutive activation of oncogenic pathways in human CRCC as evaluated after Smo and Gli1 silencing. Western blots analysis of human 786-0 cell lysates that were transiently transfected for 2 days with siRNA (siCtl, siSmo and siGli1, as indicated) at 100 nM and incubated with the antibodies against non-phosphorylated GSK-3 (GSK-3), phospho-GSK-3 (P-GSK3), non-phosphorylated Akt (Akt), phospho-Akt (P-Akt), non-phosphorylated NF-κB (NF-κB), phospho-NF-κB (P-NF-κB), non-phosphorylated Erk1/2 (Erk1/2), phospho-Erk1/2 (P-Erk1/2) and corresponding β-actin, as indicated. The gels shown are representative of at least 3 independent experiments. [file 1476-4598-8-123-S8.TIFF]
