# Supplementary material for: The sonic hedgehog signaling pathway is reactivated in human renal cell carcinoma and plays orchestral role in tumor growth
Source: Mol Cancer. 2009 Dec 16;8:123. doi: 10.1186/1476-4598-8-123 (PMC2803450; doi:10.1186/1476-4598-8-123)
Supplement: Additional file 9 — Primer pairs for the quantitative measurements of gene expression. Table showing primer pairs for the quantitative measurements of gene expression. [file 1476-4598-8-123-S9.DOC]

| Gene name | Forward primer (5’-3’) | Reverse primer (5’-3’) |
| --- | --- | --- |
| Ptch1 | TCTCGGATCATTGTGATGGA | AGGCTCAGCACTAGGCATGT |
| Smo | CAACCTGTTTGCCATGTTTG | CTCTTCTTGATCCGCTTTGG |
| Gli1 | GTGCAAGTCAAGCCAGAACA | ATAGGGGCCTGACTGGAGAT |
| Gli2 | TTTATGGGCATCCTCTCTGG | AAGGCTGGAAAGCACTGTGT |
| Gli3 | CTTTGCAAGCCAGGAGAAAC | TGTTGGACTGTGTGCCATTT |
| SHH | GCTCGGTGAAAGCAGAGAAC | CCAGGAAAGTGAGGAAGTCG |
| pCMV6-XL5 | GTGAAACCCCGTCTCTACCA | CTATTGGGAACCAAGCTGGA |

**Table S1.** Primer pairs for the quantitative measurements of gene expression.
